# What’s the Optimal Lipids Level for Dialysis Patients? A Cohort Study from a Chinese Dialysis Center in a University Hospital

**DOI:** 10.1371/journal.pone.0167258

**Published:** 2016-12-16

**Authors:** Wen- Ling Yang, Xue-Yan Zhu, Ning Zhu, Chun-Yan Su, Qing-Feng Han, Tao Wang, Ai- Hua Zhang

**Affiliations:** 1 Department of Nephrology, Peking University Third Hospital, Beijing, People’s Republic of China; 2 Department of Nephrology, Jilin medical university, Jilin province, People’s Republic of China; Chang Jung Christian University, TAIWAN

## Abstract

**Background:**

With lipid level being a major contributing factor for cardiovascular health, the high cardiovascular mortality among dialysis patients has raised substantial concerns in regard to the optimal lipid level in these patient population.

**Objective:**

To explore the optimal lipid level for the survival of dialysis patients.

**Methods:**

The lipid profile was measured for each patient. All participants were followed throughout the course of the study. Cox proportional hazards analysis was performed to analyze the prognostic value of lipid level on the survival of these patients.

**Results:**

In our study that included 311 stable maintenance dialysis patients, 54.98% of the participants had LDL-C level ≥100 mg/dl and 82.91% of the patients with triglycerides ≥200 mg/dl had non-HDL level ≥130 mg/dl. During the follow-up period of 48.0 (18.0, 55.5) months, 149 (47.91%) participants died. Among those who died, 59 patients died of cardiovascular disease (CVD) and 33 patients died of ischemic CVD (12.0, 4.7, and 2.7 events per 100 patient-years, respectively). Patients with LDL-C 100–130 mg/dl or non-HDL 130–160 mg/dl had a lower all-cause mortality rate than those who did not meet these criteria. After adjusting for the traditional and ESRD-related risk factors, non-HDL was found to be the independent risk factor for the all-cause mortality. Compared to those patients with non-HDL 130–160 mg/dl, patients with non-HDL <100 mg/dl, 100–130 mg/dl, 160–190 mg/dl, or ≥190 mg/dl all had higher all-cause mortality: HR (95% CI) 3.207 (1.801, 5.713), 2.493 (1.485, 4.184), 2.476 (1.423, 4.307), and 1.917 (1.099, 3.345), respectively. There were no differences in nutrition, comorbidity, and inflammation indices among the patients with different non-HDL groups. However, patients with non-HDL of 130–160 mg/dl had the lowest corrected calcium and calcium phosphate product values as compared with other non-HDL groups.

**Conclusion:**

Our study demonstrated that non-HDL 130–160 mg/dl might be the most appropriate lipid level in our dialysis patients. Our follow-up data also showed that patients with higher lipid level had poorer prognosis, just as in the general population.

## Introduction

A number of studies have shown that patients with end-stage renal disease (ESRD) have high cardiovascular morbidity and mortality [1–9]. Dyslipidemia, as a traditional cardiovascular risk factor, is an important “criminal” of atherosclerotic diseases in the general population [1–3]. According to the adult treatment panel III of high blood cholesterol (ATP III) [1], patients with different cardiovascular risk levels should achieve different lipid targets. The Kidney Disease Outcome Quality Initiative (K/DOQI) [2] and European Society of Cardiology (ESC) guidelines [3] recommended that LDL-C level of patients with chronic kidney diseases (CKD) stage 5 should be maintained at <100 mg/dl and <70 mg/dl respectively due to their high cardiovascular risk. However, some literatures demonstrated that dialysis patients with higher lipid level actually had better outcomes, which was called the ‘reverse epidemiology’ [4–9]. Since the results of recent large clinical trials [10–13] did not demonstrate the expected benefit of lowering LDL-C with statins in hemodialysis patients, the 2013 clinical practice guidelines for lipid management in CKD patients [4] suggested that statins or statin/ ezetimibe combination should not be initiated in adults with dialysis-dependent CKD; however, for patients already receiving statins or statin/ ezetimibe combination at the time of dialysis initiation, the guideline suggested that these agents be continued. But it gave no lipid targets [4]. As a result, the optimal lipid level for dialysis patients remains unclear [1–4, 14], and the significance of statins therapy for dyslipidemia in dialysis patients was still in disputes [1–14]. Therefore, this study aimed to find out the optimal lipid level and its effect on the mortality of stable dialysis patients.

## Materials and Methods

### Study design and population

All stable ESRD patients on maintenance dialysis who had been dialyzed in our center for more than one month before December 2008 were enrolled. Patients with acute sickness such as infection, congestive heart failure, acute coronary syndrome, symptomatic arrhythmia, active autoimmune diseases, severe lung diseases, or any other acute conditions were excluded from the study. Hospitalized or perioperative patients, patients who suffered from trauma or untreated malignancy, patients with life expectancy less than one year, and those who didn’t sign their consent to this study were also excluded. The fasting lipid profile and other biochemistry items were measured by Olympus AU2700 auto-analyzer (Olympus, Japan) as a clinical routine. To convert from mg/dl to mmol/l, multiply total cholesterol (TC), high density lipoprotein (HDL-C), low density lipoprotein- cholesterol (LDL-C) values by 0.02586 and multiply triglycerides (TG) values by 0.01129.

The traditional cardiovascular risk factors were investigated by a survey to compute the 10 years atherosclerosis risk with Framingham cardiovascular risk scoring (FCRS) algorithm previously published in ATP III [1]. Each patient’s cardiovascular risk is categorized as high (>20%), moderate (10%-20%) or low (<10%). The survey also included items about whether or not the patients were taking lipid-lowering drugs.

Previous major cardiovascular comorbid conditions were either self-reported or based on the presence of at least one hospitalization with a primary or secondary diagnosis of ischemic heart disease, cerebrovascular disease, or arterial disease, as affected by a cardiovascular disease (CVD). The Charlson Comorbidity Index (CCI) was calculated for each patient by summing the assigned weights for all comorbid conditions [15, 16].

All these patients were followed until Oct. 2013. The primary endpoint of the study was death from any cause. The secondary endpoint was cardiovascular mortality or ischemic (atherosclerosis) cardiovascular death.

All participants provided written informed consent, and the study was approved by the Medical Ethics Committee of Peking University Third Hospital.

### Statistical analysis

Statistical evaluation was performed using the SPSS statistical software program (version 21.0: SPSS, IBM corp., USA). The normally distributed variables were expressed as mean ± standard deviation (SD), while non-normally distributed variables were expressed as median (25% quartile, 75% quartile). Frequency distributions were provided for categorical variables. Mortality differences among lipids and other categories were determined with chi-square test. The mean difference was determined with t-test or one-way analysis of variance (ANOVA) for the comparison of two or three groups respectively. Bivariate correlation analysis was performed when necessary.

Survival curves were generated using the Kaplan-Meier method. All-cause mortality, cardiovascular mortality, and ischemic cardiovascular death related to the lipid levels were evaluated by multivariate Cox proportional hazards model analysis adjusted by the traditional and ESRD-related risk factors, which came from the results of single covariate analysis. The models for the mortality outcomes were censored only at death or the end of follow-up (withdrawal from the current dialysis mode, departure from the center, or at the terminating time of the study). We calculated hazard ratios (HR) and 95% confidence intervals (CI) of HR. The category 0 was the reference for the covariates.

All statistical tests were 2 sided. *P* value less than 0.05 was considered statistically significant.

## Results

### Demographic characteristics

There were 340 potentially eligible patients on maintenance dialysis. A total of 29 patients were excluded, of whom 24 had incomplete data and 5 refused to participate in the study. As a result, a total of 311 patients were enrolled in the study, including 132 patients on hemodialysis (HD, 42.44%) and 179 patients on continuous ambulatory or intermittent peritoneal dialysis (PD). The demographic characteristics of the participants were listed in Table 1. The age and dialysis vintage on enrollment averaged 59.75 years old and 33.88 months respectively. Among these patients, 48.55% (n = 151) were male. The primary diseases of ESRD were chronic glomerulonephritis (31.51%, n = 98), diabetic nephropathy (26.05%, n = 81), chronic interstitial nephritis (17.36%, n = 54), hypertension nephrosclerosis (16.72%, n = 52), autosomal dominant polycystic kidney diseases (4.18%, n = 13), postoperative urinary system tumor, and others (4.18%, n = 13). The average CCI was 2.34, and 63.02% (n = 196) patients had CCI ≥2. Meanwhile, 44.37% (n = 138) and 12.86% (n = 40) had CCI ≥3 and ≥5 respectively. Among the patients in this study, 32.80% (n = 102) suffered from diabetes mellitus. Eighty-one cases (26.05%) had CHD history.

**Table 1:** Baseline characteristics comparison between the alive or deceased dialysis patients during the follow-up.

mean±SD(n)	Total	Alive	Died	Statistics	Sig.(2-tailed)
n	311	162	149		
HD,n(%)	132(42.44)	79(59.85)	53(40.15)	5.532	.019
Male,n(%)	151(48.55)	82(54.30)	69(45.70)	.577	.448
ESRD primary diseases,n(%):				31.221	<.001
CGN	98(31.51)	68(69.39)	30(30.61)		
DN	81(26.05)	23(28.40)	58(71.60)		
CIN	54(17.36)	32(59.26)	22(40.74)		
HTN	52(16.72)	26(50.00)	26(50.00)		
Other	26(8.36)	13(50.00)	13(50.00)		
Age at exam,y	59.75±13.40	54.44±13.90	65.52±10.08	-8.087	<.001
Dialysis vintage,months	33.88±40.06	33.32±40.86	34.50±39.29	-.258	.796
Ultrafiltration,l	1.32±1.23	1.42±1.29	1.21±1.15	1.468	.143
Dry weight,kg	62.29±11.81	63.17±12.09	61.33±11.47	1.357	.176
CVD risk factors count	2.56±.89	2.54±1.01	2.59±.74	-.535	.593
SBP, mmHg	140.13±23.55	139.21±21.35	141.13±25.76	-.709	.479
DBP,mmHg	76.88±12.53(302)	78.43±11.87	75.24±13.03	2.219	.027
Hemoglobin,g/l	113.52±15.48	113.36±14.12	113.69±16.90	-.188	.851
Potassium,mmol/l	4.64±.80	4.81±.72	4.45±.83	3.999	<.001
Bicarbonate,mmol/l	23.15±5.28(307)	22.75±5.45	23.60±5.06	-1.408	.160
Albumin,g/l	39.38±4.09(309)	40.96±3.06	37.64±4.37	7.662	<.001
Creatinine,umol/l	904.02±246.37	959.79±248.88	842.98±229.30	4.285	<.001
Uric acid,umol/l	418.94±94.16(308)	421.48±99.01	416.12±88.71	.498	.619
Cholesterol,mmol/l	4.79±1.14	4.71±1.00	4.88±1.28	-1.324	.187
Triglycerides,mmol/l	2.21±1.38	2.17±1.29	2.26±1.47	-.563	.574
HDL-C,mmol/l	1.10±.37	1.05±.33	1.16±.41	-2.501	.013
LDL-C,mmol/l	2.80±.95	2.72±.79	2.89±1.10	-1.590	.113
us-CRP,mg/l	4.67±7.66(209)	3.31±4.09	5.93±9.71	-2.582	.011
Transferrin saturation,%	32.02±13.53(283)	33.44±14.20	30.45±12.62	1.869	.063
Ferritin,ng/ml	440.41±336.19(296)	409.26±293.62	474.19±375.11	-1.649	.100
iPTH,pg/ml	187.04±221.13(303)	203.88±266.25	168.45±155.81	1.395	.164
Ca×P,mmol^2^/l^2^	4.21±1.47(307)	3.91±1.34	4.55±1.54	-3.934	<.001
Phosphate,mmol/l	1.77±.51	1.85±.55	1.68±.45	2.934	.004
CCI	2.34±1.79	1.49±1.50	3.26±1.62	-10.037	<.001
FCRS risk category,n(%)				35.623	<.001
>20%	184(59.16)	70(38.05)	114(15.49*)		
10–20%	29(9.33)	21(72.41)	8(6.90*)		
<10%	98(31.51)	71(72.45)	27(6.89*)		

Note: HD, Hemodialysis. ESRD, end-stage renal diseases. CGN, Chronic glomerulonephritis. DN, Diabetic nephropathy. CIN, Chronic interstitial nephritis. HTN, Hypertension nephrosclerosis. CVD, Cardiovascular diseases. SBP systolic blood pressure; DBP diastolic blood pressure. HDL-C, high density lipoproteins- cholesterol. LDL-C, low density lipoproteins-cholesterol. To convert from mg/dl to mmol/l, multiply TC, HDL-C, LDL-C values by 0.02586 and multiply triglycerides values by 0.01129. us-CRP, ultra-sensitive C reactive protein. iPTH, intact parathyroid hormone. Ca×P, corrected calcium phosphate product. CCI, The Charlson comorbidity index. FCRS, Framingham cardiovascular risk scoring algorithm, previously published in ATPIII to compute the 10 years cardiovascular atherosclerosis risk.

* Death outcomes were expressed as number (incidence rate per 100 patient-years).

### Dialysis related parameters

As listed in Table 1, the ultrafiltration volume and dry body weight averaged 1.42 L and 62.29 kg respectively. As for HD patients, one hundred and four cases (78.79%) had three dialysis sessions per week, twenty-six patients (19.70%) had two sessions per week, and two patients had hemodialysis treatment once every two weeks for more than one year. Meanwhile 87.12% of the HD patients (n = 115) had four hours dialysis session. Ten patients had 4.5 hours dialysis session, and the remaining patients had 3–3.5 hours per treatment. The median blood flow, ultrafiltration volume, and corrected single-pool urea clearance index (KT/V_urea_) was 275ml/min, 2.50 kg per session and 1.38 respectively. The mean blood pressure and heart rate pre- vs post-dialysis were 151/76 vs 137/71 mmHg and 76 vs 73 beats per minute respectively. As for PD patients, all were on glucose lactate-buffered dialysis solution (Dianeal, Baxter). The infused dialysis solution volume was 6000 (4850, 8000) ml/d and the mean ultrafiltration volume was 0.45 L/d. The corrected KT/V_urea_ value for peritoneal dialysis patients was 1.90±.50.

### Lipid profile of the dialysis patients

The biochemistry characteristics of the patients were listed in Table 1. The average TC, TG, LDL-C, HDL-C and non-HDL were 4.79mmol/l, 2.21mmol/l, 2.80mmol/l, 1.10mmol/l and 3.69mmol/l respectively.

**Fig 1:**
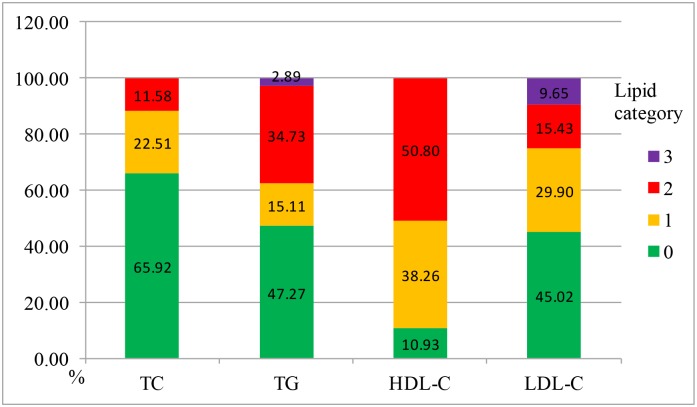
The lipid categories of dialysis patients according to the criteria in ATP III guideline. Note: Lipid categories according to ATP III: TC, total cholesterol: 0: optimal, <200 mg/dl; 1: borderline high, 200–239 mg/dl; 2: high, ≥240 mg/dl. TG, Triglycerides: 0: optimal, <150 mg/dl; 1: borderline high, 150–199 mg/dl; 2: high, 200–499 mg/dl; 3: very high, ≥500 mg/dl. LDL-C, low density lipoprotein cholesterol: 0: optimal, <100 mg/dl; 1: near optimal, 100–129 mg/dl; 2: borderline high, 130–159 mg/dl; 3: high or very high, ≥160 mg/dl. HDL-C, high density lipoprotein: 0: ≥ 60 mg/dl; 1: 40–60 mg/dl; 2: <40 mg/dl.Of all the patients, thirteen cases (4.18%) were taking statin drugs, and only one patient was taking fenofibrate.

According to the cut-points in ATP III [1], the lipid parameters were classified as optimal in 65.92% of the participants (n = 205) with cholesterol level <200 mg/dl, 47.27% (n = 147) with triglycerides<150 mg/dl, and 45.02% (n = 140) with LDL-C <100 mg/dl. It was classified as high or very high in 37.62% of the subjects (n = 117) with triglycerides level ≥200 mg/dl, 11.58% (n = 36) with cholesterol ≥240 mg/dl, and 9.65% (n = 30) with LDL-C ≥160 mg/dl. In addition, 50.80% (n = 158) of the participants had a HDL-C level lower than 40 mg/dl (Fig 1). In other words, 171 cases (54.98%) had LDL-C level ≥100 mg/dl (target defined by K-DOQI [2]) and 269 cases (86.50%) had LDL-C level ≥70 mg/dl (target defined by ESC [3]). For those patients with high triglycerides (≥200 mg/dl), 82.91% (n = 97) had higher non-HDL level according to target defined by K-DOQI [2], while 97.44% (n = 114) had higher non-HDL levels according to target defined by ESC [3]. While for those with fit LDL-C and TG ≥200 mg/dl, 50.00% (n = 20) and 72.73% (n = 8) had non-HDL level higher than the target defined by K-DOQI [2]) and ESC [3] respectively. According to ATP III [1], 39.55% LDL-C (123/311) and 47.27% non-HDL (26/55) levels were higher than the targets.

### Lipids and mortality

During the follow-up period of 48.0 (18.00, 55.50) months, 149 (47.91%) participants died (53 cases on HD (40.15%) and 96 cases on PD (53.63%)). Among those who died, 59 (39.60%) patients died of CVD causes and 33 (22.15%) patients died of ischemic CVD causes. Thirteen patients had kidney transplantation. Fourteen patients were transferred from one type of dialysis modality to the other, and 25 patients were lost during follow-up due to the departure to other dialysis units. Compared with hemodialysis patients, those on peritoneal dialysis had more deaths (χ^2^ = 5.532, p = .019).However, we found no difference in CVD mortality or ischemic CVD mortality between HD and PD patients.

The mean dialysis vintage at death was 65.05 months. Cardiac death (including sudden death and congestive heart failure) accounted for 19.46% (n = 29). Cerebrovascular diseases including ischemic (n = 12) and hemorrhagic cases accounted for 16.78% (n = 25). Sepsis accounted for 21.48% (n = 32). Twenty-eight cases died of multiple organ dysfunction syndromes (MODS, 18.79%). Thirty-five cases (23.49%) died of other causes including digestive system diseases (n = 12, 8.05%), such as upper gastrointestinal tract bleeding, severe pancreatitis and digestive tract perforation, malignancy (n = 8, 5.37%), peripheral vascular diseases (n = 4), and unknown causes.

**Fig 2:**
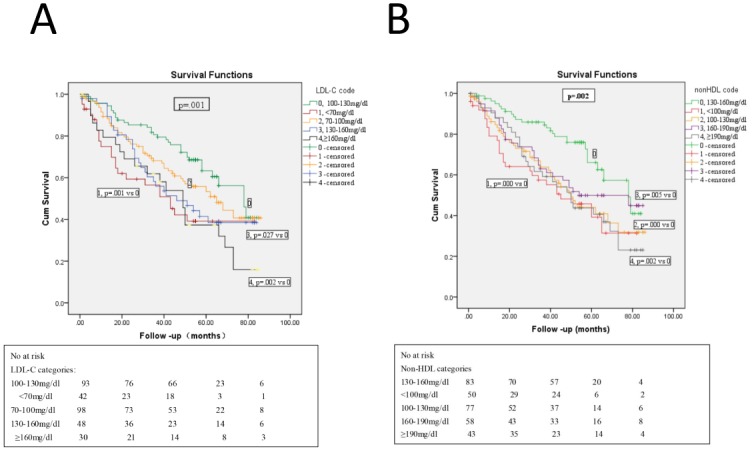
Survival curve stratified by lipid categories in maintenance dialysis patients. Note: (A) Kaplan- Meier curves for survival by the LDL-C categories. The person was categorized according to the level of LDL-C as 0 (100–130 mg/dl), 1 (<70 mg/dl), 2 (70–100 mg/dl), 3 (130–160 mg/dl) and 4 (≥160 mg/dl). The all-cause mortality risk was the lowest in patients with LDL-C category 100-130mg/dl. (B) Kaplan- Meier curves for survival by the non-HDL categories. The person was categorized according to the level of non-HDL as 0 (130–160 mg/dl), 1 (<100 mg/dl), 2 (100–130 mg/dl), 3 (160–190 mg/dl), and 4 (≥190 mg/dl). The all-cause mortality risk was the lowest in patients with non-HDL category 130-160mg/dl.

Table 1 listed the biochemical comparison between the alive and deceased patients. The patients who died seemed to be older, and have lower levels of DBP. Their serum potassium, albumin, creatinine, and phosphate level was also lower as compared with that of the patients alive. Meanwhile, the patients who died had higher CCI, ultra-sensitive C reactive protein (us-CRP), and corrected calcium phosphate product (abbreviated as Ca×P). Only HDL-C level among the measured lipids seemed to have significant difference between the alive and deceased patients. We classified lipids into categories as the following: TC, 0: 160–200 mg/dl, 1: <160 mg/dl, 2: 200–239 mg/dl, 3: ≥240 mg/dl; LDL-C, 0: 100–130 mg/dl, 1: < 70 mg/dl, 2: 70–100 mg/dl, 3: 130–160 mg/dl, 4: ≥160 mg/dl; non-HDL, 0: 130–160 mg/dl, 1: < 100 mg/dl, 2: 100–130 mg/dl, 3: 160–190 mg/dl, 4: ≥190 mg/dl; TG, 0: 150–200 mg/dl, 1: <150 mg/dl, 2: 200–500 mg/dl, 3: ≥500 mg/dl. The all-cause mortality risk related to LDL-C and non-HDL categories was shown as Kaplan-Meier plot in Fig 2. There was significant difference in the survival rate of patients with different LDL-C categories (log rank test, χ^2^ = 17.883, p = .001) or non-HDL categories (log rank test, χ^2^ = 17.408, p = .002); however, there was no significant difference among different TC categories. The pair-wise comparison of the all-cause mortality risk related to LDL-C (Fig 2A) or non-HDL (Fig 2B) categories was also shown in Fig 2. When we take the FCRS cardiovascular risk into consideration, we found that there was also significant difference in the all-cause mortality among LDL-C categories (log rank test, χ^2^ and the relative p value was 12.937, .012; 9.697, .046 respectively), and non-HDL categories (log rank test, χ^2^ and the relative p value was 9.688, .046; 16.242, .003 respectively) whether or not the FCRS risk is low or high. The mortality difference among the different LDL-C or non-HDL categories could not be computed when the FCRS risk was between 10–20% because there was no death event in some of the categories.

The mortality of any cause, CVD, and ischemic CVD related to lipids (LDL-C, non-HDL, and TC categories as classified previously) were evaluated by multivariate Cox proportional hazards model analysis. The covariates included dialysis mode, age at test, albumin, serum creatinine, serum phosphate, Ca×P, DBP, systolic blood pressure (SBP), serum potassium code (0: 3.50–5.499 mmol/l; 1: <3.50; 2: 5.50–5.999 mmol/l; 3: 6.00–6.499 mmol/l; 4: ≥6.50 mmol/l), FCRS categories (0: <10%; 1:10–20%; 2:>20%), and CCI (Model 1). Non-HDL was the only lipid category that was associated with all-cause mortality (P = .001). With category zero (130-160mg/dl) patients as the reference, non-HDL categories 1–4 patients all had higher risk of all-cause mortality (HR (95% CI): 3.207 (1.801, 5.713), 2.493 (1.485, 4.184), 2.476 (1.423, 4.307), 1.917 (1.099, 3.345) respectively). Higher CCI value was related to an increase of all-cause mortality, CVD or ischemic CVD death (HR (95%CI): 1.401 (1.264, 1.554), 1.384 (1.203, 1.593) and 1.438 (1.147, 1.801) respectively). Higher SBP was associated with increased risk for CVD death (HR (95% CI): 1.012 (.999, 1.025), p = .068). Lower serum albumin was associated with increased all-cause mortality (HR (95% CI): .925 (.890, .962)). Schoenfeld residuals test demonstrated that all the covariates didn’t change with time.

Since CCI was shown to be an important confounding factor, we perform Cox-regression survival analysis stratified by CCI (0: 0–3; 1:≥3, Model 3). The result was similar to that with CCI as covariates. Non-HDL was associated with the all-cause mortality (p = .020), while higher SBP increased the risk for all-cause mortality (HR (95% CI): 1.010 (1.002, 1.017)) and CVD death (HR (95% CI): 1.013 (1.001, 1.026)). Compared with patients in non-HDL category zero (130–160 mg/dl), patients in non-HDL category 1–4 all had higher risk of all-cause mortality (HR (95% CI): 2.563 (1.436, 4.574), 1.981 (1.167, 3.363), 2.108 (1.207, 3.682), 2.019 (1.148, 3.549) respectively) (Table 2). Lower serum albumin was associated with increased all-cause mortality (HR (95% CI) .913 (.879, .949)) and ischemic CVD death (HR (95% CI) .927 (.857, 1.003), P = .061). The increase in all-cause mortality rate per 1000 person-years attributable to every 1 g/l decrease in ALB was 87 stratified and 75 unstratified. Older age at enrollment also increased the all-cause mortality risk (HR (95% CI): 1.022 (1.004, 1.041)).

**Table 2:** The Cox- regression survival analysis result for all- cause mortality related to non-HDL.

All-cause mortality	Model 1		Model 2		Model 3		Model 4	
	HR (95%CI)	P value	HR (95%CI)	P value	HR (95%CI)	P value	HR (95%CI)	P value
Non-HDL (mg/dl)		.001		.001		.020		.020
130–160	1		1		1		1	
<100	3.207(1.801,5.713)	<.001	3.207(1.801,5.713)	<.001	2.563(1.436,4.574)	.001	2.563(1.436,4.574)	.001
100–130	2.493(1.485,4.184)	.001	2.493(1.485,4.184)	.001	1.981(1.167,3.363)	.011	1.981(1.167,3.363)	.011
160–190	2.476(1.423,4.307)	.001	2.476(1.423,4.307)	.001	2.108(1.207,3.682)	.009	2.108(1.207,3.682)	.009
> 190	1.917(1.099,3.345)	.022	1.917(1.099,3.345)	.022	2.019 (1.148,3.549)	.015	2.019 (1.148,3.549)	.015

Note: CI, confidence interval. Because only non-HDL categories among the lipids were shown to be associated with the all-cause mortality, as a result, the table only listed the devotion of non-HDL categories.

Model 1: The adjusted covariates included dialysis mode, age at enrollment, albumin (ALB), serum creatinine, serum Phosphate, corrected calcium phosphate product (Ca×P), diastolic blood pressure, systolic blood pressure (SBP), serum potassium code (0: 3.50–5.499 mmol/l; 1: <3.50 mmol/l; 2: 5.50–5.999 mmol/l; 3: 6.00–6.499 mmol/l; 4: ≥6.50 mmol/l), FCRS (Framingham cardiovascular risk scoring) risk categories (0, <10%; 1, 10–20%; and 2, >20%), Charlson comorbidity index (CCI), and lipids categories (LDL-C (0: 100–130 mg/dl;1:< 70 mg/dl; 2: 70–100 mg/dl; 3: 130–160 mg/dl; 4: ≥160 mg/dl); non-HDL (0: 130–160 mg/dl; 1:< 100 mg/dl; 2: 100–130 mg/dl; 3: 160–190 mg/dl; 4: >190 mg/dl); total cholesterol (0: 160–200 mg/dl; 1: <160 mg/dl; 2: 200–239 mg/dl; 3: ≥240 mg/dl)), with reference of category zero.

Model 2: The covariates included sex, smoking status and all the above except FCRS risk categories.

Model 3: The Cox regression survival analysis was stratified by CCI (0: <3; 1: ≥3). The covariates included all the above in model 1 except CCI.

Model 4: The analysis was stratified by CCI (0:<3; 1:≥3). The covariates included all the above in model 2 except CCI.

Without the covariate of FCRS categories (Model 2 unstratified by CCI and Model 4 stratified by CCI), the Cox-Regression results, adjusted for all other covariates plus the component factors of FCRS such as smoking status and sex, also demonstrated that non-HDL was an independent risk factor for all-cause mortality (Table 2, p <.05).

Table 3 listed the demographic and biochemical comparison among the different non-HDL categories. The patients in non-HDL category zero had the lowest Ca×P and corrected calcium as compared to the patients in other categories. However, the pair-wise comparison demonstrated that the difference in Ca×P reached statistically significant only between the non-HDL category 2 and 0. For corrected calcium, there was significant difference only between the non-HDL category 4 and 0. There was no difference in age at enrollment, dry body weight, serum albumin, us-CRP, ferritin, CCI, phosphate, hemoglobin, blood pressure, iPTH, and serum creatinine among the patients in different non-HDL categories.

**Table 3:** Patients characteristics by non-HDL categories.

Non-HDL(mg/dl)mean ± SD(n)	130–160	<100	100–130	160–190	≥190	Statistics	P value
N	83	50	77	58	43		
Age at exam	56.73±13.50	62.78±12.10	60.88±13.64	58.28±14.71	62.00±11.35	2.346	.055
HD,n(%)	41(49.40)	35(70.00)	45(58.44)	9(15.52)	2(4.65)	67.606	<.001
Male,n(%)	44(53.01)	35(70.00)	43(55.84)	19(32.76)	10(23.26)	28.315	<.001
Dry body weight,kg	64.12±11.94	61.96±10.56	61.50±10.79	62.43±12.65	60.17±12.88	.954	.433
Dialysis vintage at test,months	32.92±40.01	52.64±52.40	40.16±42.70	22.41±27.38^#^^δ^	18.19±16.43*^##^^δ^	8.526	<.001
Albumin,g/l	40.18±3.78(82)	39.39±4.46(49)	39.21±4.19	39.30±4.30	38.23±3.58	1.686	.153
CCI	2.20±1.83	2.48±1.74	2.16±1.59	2.26±1.86	2.86±1.98	1.343	.254
Corrected calcium posphate product,mmol^2^/l^2^	3.82±1.30(82)	4.28±1.66(49)	4.46±1.50*	4.16±1.41	4.48±1.46	2.442	.047
Corrected Calcium,mmol/l	2.26±.79(82)	2.45±.84(49)	2.52±.84	2.47±.82	2.73±.70*	2.534	.040
Phosphate,mmol/l	1.75±.51(82)	1.80±.49	1.86±.60	1.74±.45	1.66±.41	1.176	.321
Follow-up duration,months	47.31±21.37	34.94±24.30*	37.82±25.01*	44.74±25.23^#^	43.49±24.79	2.917	.022
Hemoglobin,g/l	112.59±12.90	111.92±15.05	111.27±15.96	116.45±17.98	117.20±15.46	1.757	.138
WBC	7.02±1.67	6.18±2.11*	7.01±2.33^#^	7.76±2.23*^##^^δ^	7.78±2.22^##^	5.026	.001
DBP,mmHg	77.44±10.82(78)	75.61±12.31(48)	76.11±14.10(75)	77.72±12.83	77.48±12.75	.319	.865
SBP,mmHg	140.77±20.08	146.39±22.21	140.94±27.02	138.96±25.66	131.75±19.82	2.360	.053
iPTH,pg/ml	191.84±309.03(80)	213.45±212.83	162.28±144.70(76)	212.99±214.54(54)	158.61±141.29	.787	.534
Ultrafiltration,l	1.43±1.32	1.94±1.28	1.63±1.23	.67±.86*^##^^δδ^	.70±.62*^##^^δδ^	18.047	<.001
us-CRP,mg/l	3.59±5.37(49)	3.82±5.14(26)	5.19±8.67(48)	3.72±5.01(46)	7.04±11.47(40)	1.019	.402
Ferritin,ng/ml	386.63±273.05(79)	423.39±350.98(48)	419.14±303.05(75)	502.77±340.97(54)	522.75±453.53(40)	1.691	.152
Urea nitrogen,mmol/l	25.96±6.69	26.47±5.86	25.49±6.49	23.38±5.94*^#^	20.70±5.18**^##^^δδ^^Δ^	7.350	<.001
Bicarbonate,mmol/l	22.43±5.62(80)	20.62±5.00(50)	22.39±5.38(77)	24.90±4.52^##^^δ^(57)	26.47±3.10**^##^^δδ^	16.106	<.001
Potassium,mmol/l	4.72±.68	4.75±.80	4.68±.89	4.60±.91	4.34±.58*	3.321	.012
Creatinine,umol/l	890.51±266.24	944.80±222.96	935.65±199.51	897.72±294.50	834.26±230.07	1.893	.115

Note: HD, hemodialysis. Apo, apoliporotein. CCI, The Charlson comorbidity index. WBC, white blood cell; DBP, diastolic blood pressure. SBP, systolic blood pressure. iPTH, intact parathyroid hormone. us-CRP, ultra-sensitive C reactive protein.

*, p< .05,

**, p< .001 compared with non-HDL category zero;

^#^, p< .05,

^##^, p< .001compared with non-HDL category 1;

^δ^, p< .05,

^δδ^, p< .001 compared with non-HDL category 2;

^Δ^, p< .05 compared with non-HDL category 3.

## Discussion

Our study found that those patients with higher lipid level generally have poorer prognosis, similar to the general population. However, our results also showed that patients with too low level of lipid, which was recommended as ideal or optimal by guidelines, have poor prognosis. The LDL-C 100–130 mg/dl and the non-HDL 130–160 mg/dl seemed to be the most appropriate lipid levels for dialysis patients because these patients had the lowest all-cause mortality rate.

### Prevalence of dyslipidemia

According to K-DOQI and ESC lipid guidelines, 54.98% and 86.50% respectively of the dialysis patients in our center had LDL-C higher than the recommended targets. The former percentage (54.98%) was similar to that reported by the NKF Work Group on HD study [2] (55.7%), but lower than that of the DMM PD study [2] (78.6%) and NHANES survey [17] in CKD 1–4 stages patients (approximately 70%). The ratio of high LDL-C according to ATP III guideline in our study (39.55%) was far lower than that of the NHANES survey [17] (81%).

In our study, 37.62% of the patients had triglycerides (≥200 mg/dl) classified as high or very high, which is lower than that reported by Kasiske *et al*. [18] (45%), but higher than that by NKF work group [2] (28.1%). Approximately 50.80% of HDL-C from our study was <40 mg/dl, and this percentage was higher than that presented in the NHANES study [17] (20% or so) but similar to that in the CHOICE study (45%-48%) [19]. Kasiske *et al*. [18] reported that approximately 50% of patients had HDL-C < 35 mg/dl.

For those with TG ≥200 mg/dl, 82.91% had non-HDL ≥130 mg/dl, and this was higher than that reported by the NKF Work Group [2] (22.7%). The enormous difference observed between our study and that of the NKF Work Group HD study could be due to some differences in non-HDL application premises. The common is that both figures did not take LDL-C into consideration in computing. What is different was the denominator. Note that, if the same criteria was used in our study, 6.43% (n = 20) with normal LDL (<100 mg/dl) would require treatment based on TG ≥200 mg/dl and non-HDL ≥130 mg/dl, similar to that in the DMM HD study and PD study (5.4%) [2]. We believe it is logical to use the higher- than- target ratio of non-HDL among those who has normal LDL-C (<100 mg/dl) and TG ≥200 mg/dl to evaluate the lipid status.

On the whole, dyslipidemia of our dialysis center presented as similar to or even better than that reported in the literatures [2, 4–9, 18].

### Lipids and mortality

Few studies [4, 12, 20–21] had examined the association between hyperlipidemia and mortality in dialysis-dependent patients. Although dyslipidemia is a well established traditional risk factor for mortality in the general population, it did not appear to be independently associated with increased risk for all-cause mortality in dialysis patients. In contrast, some literatures hinted that high cholesterol was associated with lower mortality in patients on dialysis [12, 21–22]. Our results support that those patients with high lipid level had poor prognosis similar to the general population. However, our results also suggested that patients with too low lipid level, which was recommended as ideal or optimal lipids level by guidelines, had poor prognosis. We believe that too low level or what is recommended as ideal lipid level by guidelines might be a sign of malnutrition or other comorbidities in the studied subjects.

The LDL-C of 100–130 mg/dl (n = 93, 29.90%) or the non-HDL of 130–160 mg/dl (n = 83, 26.69%) seemed to be the most appropriate lipid level for dialysis patients because patients in this category had the lowest all-cause mortality. This finding is similar to the current clinical guideline in Japan which recommended that LDL-C be <120 mg/dl, or non-HDL be <150 mg/dl as an alternative target in patients with chronic kidney disease [23]. Based on the subgroup analysis of 4D, it may be possible to recommend that statins treatment be initiated if LDL-C is higher than 145 mg/dl in hemodialysis patients.[23] However, Kim *et al* demonstrated that high serum LDL-C (≥100mg/dl) at the time of HD commencement was a significant independent risk factor for the composite outcome of all-cause mortality and CV events in 867 incident HD patients during the early stages of dialysis, even after adjusting for age, gender, diabetes mellitus, preexisting CV disease, albumin, and hs-CRP. [24]

Whether different FCRS risk patients had different prognosis when they had the same lipid level? Our result indicated that lipid level was the independent risk factor for prognosis regardless of the FCRS risk level. Adjusted for the cardiovascular risk categories and other covariates, non-HDL of 130–160 mg/dl seemed to have the lowest all-cause mortality ratio when compared with other non-HDL categories. This indicates that the management of dyslipidemia in dialysis patients may not be as confusing as once thought. The lipid targets for dialysis patients might not be different in high or low CVD risk patients as reported in ATP III.

Why did the patients with non-HDL of 130–160 mg/dl have the lowest mortality? Our result demonstrated that although there was no difference in nutrition, iPTH, comorbidity and inflammation indices among the patients in different non-HDL categories, the patients in non-HDL category zero had the lowest corrected calcium and calcium phosphate product. Higher Ca×P value may predispose vascular or soft tissue calcification and higher mortality. We thus speculated that the lower Ca×P value might have contributed to the lower all-cause mortality for patients in non-HDL category zero. Several cross-sectional studies have found an association between deficiencies in serum vitamin D and an atherogenic lipid profile such as higher non-HDL, or LDL-C level [25]. Patwardhan *et al* [26] reported that 25(OH) D seemed to show a varying relationship with HDL-C at different duration of sunlight exposure. At lower sunlight exposure (<1 h/d), serum 25(OH) D levels were positively associated with HDL-C levels (P < .05), but at higher sunlight exposure (>2 h/d), serum 25(OH) D concentrations were significantly negatively associated with HDL-C (P < .05). As we know, deficiency in vitamin D is prevalent in ESRD patients on dialysis, which interacts with iPTH and fibroblastic growth factor 23 (FGF23)/Klotho axis and devotes to lower calcium. It was demonstrated that FGF23, nuclear factor-Kb and fetuin A etc might mediate the linkage of calcium phosphate disorder and atherosclerosis [27]. However, the specific mechanism that underlies the association between the lipids and calcium was still unclear.

It is interesting to note that only non-HDL, which is equal to TC minus HDL-C, rather than LDL-C or TG appeared to be associated with the all-cause mortality. Studies suggested that elevated triglyceride or the accumulation of triglycerides-rich lipoproteins such as very low density lipoprotein and intermittent density lipoprotein (IDL) were important atherogenic lipid components [2, 5–8, 18, 22]. Nowadays, non-HDL has turned out to be a good surrogate marker of triglycerides and its remnants [1, 3, 28]. In the presence of high serum triglycerides (200–499 mg/dl, 34.73% in our center), non-HDL cholesterol will better represent the concentrations of all atherogenic lipoproteins than will LDL-C alone because most cholesterol occurring in the very low density lipoprotein fraction is contained in smaller one. Moreover, non-HDL cholesterol is highly correlated with total apolipoprotein B, which is the major atherogenic apolipoprotein. Shoji *et al*. [29] reported in an observational cohort study of 45,390 hemodialysis patients without previous history of ischemic cardiovascular diseases in Japan that the one-year incidence of myocardial infarction and cerebral infarction were positively associated with non-HDL level. Echida *et al*. [30] reported that non-HDL was an independent predictor of cardiovascular mortality in 259 HD patients (HR 1.015, 95% CI 1.004–1.025, p = 0.0083, area under the curve (AUC) 0.62416; p = 0.0366; cutoff value 111.0 mg/dl).

Undoubtedly, the cardiovascular mortality of dialysis patients is extremely high as compared to that of the general population. However, we did not found a strong association between lipids and CVD or ischemic CVD death in our study. We believe that the CVD mortality here, like other reports, incorporated deaths that would be caused by disorders in which dyslipidemia may not be a major pathogenic factor such as cardiomyopathy, cardiac arrest, pulmonary edema, and arrhythmias. For example, left ventricular hypertrophy, which is extremely common in patients with CKD, is a very strong predictor of mortality, but is largely unrelated to dyslipidemia [22]. Perhaps, this can explain the lack of strong relationship between lipids and CVD mortality in the dialysis patients. On the other hand, in our study, there were only 33 cases of ischemic CVD death, which might have also affected the result of Cox analysis. A larger scale study to elucidate the association between the lipid level and ischemic CVD mortality is warranted.

One of the limitations of this study is the use of a single baseline measurement of lipids to predict several events in the future. However, there are several precedents [31] which used a single baseline measurement to predict future multiple events. The strong association observed in the present study indicated the potential power of dyslipidemia in predicting patient outcome in dialysis patients. Additionally, our results were limited by the sample size and the innate shortcomings of an observational study. A larger scale study is needed to further solidify our findings.

## Conclusion

In this cohort of subjects on stable maintenance dialysis, attainment of LDL-C/non-HDL goal was similar to or better than that reported in the literatures. But the prevalence of dyslipidemia was still high. Similar to what was found in the general population, our follow-up data in dialysis patients supported that those with higher lipid level had poorer prognosis. Meanwhile our result also suggested that patients with too low levels of lipid (LDL-C <70 mg/dl, non-HDL <130 mg/dl), which were recommended as optimal lipid levels by current guidelines, had poor prognosis. The non-HDL of 130–160 mg/dl seemed to be the most appropriate lipid level for dialysis patients. We also found that the patients in non-HDL category (130–160 mg/dl) had the lowest corrected calcium and calcium phosphate product compared to those patients in other non-HDL categories. However, the mechanism underlying the association of lipid and calcium needs further investigation. Given the high all-cause and cardiovascular mortality rates observed in dialysis patients, the role of lipids as a modifiable risk factor should be highlighted.

## Supporting Information

S1 File 2016-09-17 PACE QUALITY REPORTS.pdf. The PACE Quality report for the figures.
(PDF)

S2 File 2016-11-18 PACE Quality Reports.pdf. The PACE quality report for the combined Fig 2.
(PDF)

S3 File PLOS ONE Clinical Studies checklist.docx. (DOCX)

S4 File STROBE_checklist_v4_combined_PlosMedicine.docx. (DOCX)

S5 File Raw data 1.sav. The raw data of this study (Part 1).
(SAV)

S6 File Raw data 2.sav. The KT/V evaluation data for patients on peritoneal dialysis. Part 2 of the raw data of this study.
(SAV)
