# Supplementary material for: What’s the Optimal Lipids Level for Dialysis Patients? A Cohort Study from a Chinese Dialysis Center in a University Hospital
Source: PLoS One. 2016 Dec 16;11(12):e0167258. doi: 10.1371/journal.pone.0167258 (PMC5161355; doi:10.1371/journal.pone.0167258)
Supplement: S1 File — The PACE Quality report for the figures. (PDF) [file pone.0167258.s001.pdf]

Figure file quality report: 2016-09-17				
Original Filename	PACE Filename	Status	Error Detail(s)	PACE Adjustments
fig 2a		✓	• No Error	
fig 2b		✓	• No Error	
Fig 1		✓	• No Error	
