# Supplementary material for: What’s the Optimal Lipids Level for Dialysis Patients? A Cohort Study from a Chinese Dialysis Center in a University Hospital
Source: PLoS One. 2016 Dec 16;11(12):e0167258. doi: 10.1371/journal.pone.0167258 (PMC5161355; doi:10.1371/journal.pone.0167258)
Supplement: S2 File — The PACE quality report for the combined Fig 2. (PDF) [file pone.0167258.s002.pdf]

PLOS

Figure file quality report: 2016-11-18				
Original Filename	PACE Filename	Status	Error Detail(s)	PACE Adjustments
Fig 2		✓	• No Error	
