# Supplementary material for: What’s the Optimal Lipids Level for Dialysis Patients? A Cohort Study from a Chinese Dialysis Center in a University Hospital
Source: PLoS One. 2016 Dec 16;11(12):e0167258. doi: 10.1371/journal.pone.0167258 (PMC5161355; doi:10.1371/journal.pone.0167258)
Supplement: S4 File — (DOCX) [file pone.0167258.s004.docx]

STROBE Statement—checklist of items that should be included in reports of observational studies

|  | | Item No. | | | Recommendation | | Page  No. | Relevant text from manuscript |
| --- | --- | --- | --- | --- | --- | --- | --- | --- |
| **Title and abstract** | | 1 | | | (*a*) Indicate the study’s design with a commonly used term in the title or the abstract | | 1 | Cohort study |
|  |  |  |  |  | (*b*) Provide in the abstract an informative and balanced summary of what was done and what was found | | 2-3 | abstract |
| Introduction | | | | | | | |  |
| Background/rationale | | 2 | | Explain the scientific background and rationale for the investigation being reported | | 4 | | introduction |
| Objectives | | 3 | | State specific objectives, including any prespecified hypotheses | | 4 | | This study aimed to… |
| Methods | | | | | | | |  |
| Study design | 4 | | Present key elements of study design early in the paper | | | | 5-6 | Study design and population |
| Setting | 5 | | Describe the setting, locations, and relevant dates, including periods of recruitment, exposure, follow-up, and data collection | | | | 5-6 | …at Peking University Third Hospital, December 2008 to March 2009, no exposure, Follow-up till …, The traditional cardiovascular risk factors were investigated with the survey form…, A previous major cardiovascular and other comorbid conditions were either self- reported or based on…, |
| Participants | 6 | | (*a*) *Cohort study*—Give the eligibility criteria, and the sources and methods of selection of participants. Describe methods of follow-up  *Case-control study*—Give the eligibility criteria, and the sources and methods of case ascertainment and control selection. Give the rationale for the choice of cases and controls  *Cross-sectional study*—Give the eligibility criteria, and the sources and methods of selection of participants | | | | 5-7 | …were enrolled except…, follow-up till …, The primary endpoint was…, The secondary endpoints were …, The models for the mortality outcomes were censored only at… |
|  |  |  | (*b*) *Cohort study*—For matched studies, give matching criteria and number of exposed and unexposed  *Case-control study*—For matched studies, give matching criteria and the number of controls per case | | | | no | No matched studies |
| Variables | 7 | | Clearly define all outcomes, exposures, predictors, potential confounders, and effect modifiers. Give diagnostic criteria, if applicable | | | | 6-7 | The primary endpoint was…, The secondary endpoints were …, no exposures, predictors include lipids and FCRS risk, potential confounders include the traditional and ESRD –related risk factors, COX proportional hazards model analysis |
| Data sources/ measurement | 8* | | For each variable of interest, give sources of data and details of methods of assessment (measurement). Describe comparability of assessment methods if there is more than one group | | | | 5-6 | fasting lipids test, …were measured with … according to … To convert from mg/dl to mmol/l, …were investigated with the survey form to compute 10 years atherosclerosis risk score with FCRS …, CCI was also calculated for each patient by… |
| Bias | 9 | | Describe any efforts to address potential sources of bias | | | | 6 | Corrected by the traditional and ESRD –related risk factors, to stratified with … |
| Study size | 10 | | Explain how the study size was arrived at | | | | No | No study size predefined. |

Continued on next page

| Quantitative variables | 11 | | Explain how quantitative variables were handled in the analyses. If applicable, describe which groupings were chosen and why | 6 | The normally distributed variables were expressed as…, …risk factors came from the results of single covariate analysis. |
| --- | --- | --- | --- | --- | --- |
| Statistical methods | 12 | | (*a*) Describe all statistical methods, including those used to control for confounding | 6-7 | ANOVA or chi-square test, Kaplan-Meier, Cox proportional hazards model analysis… |
|  |  |  | (*b*) Describe any methods used to examine subgroups and interactions | 6-7 | Bivariate correlation analysis was …, |
|  |  |  | (*c*) Explain how missing data were addressed | 6-7,11 | …withdrawal from the current dialysis style or center,…were lost follow-up due to the change to other dialysis units |
|  |  |  | (*d*) *Cohort study*—If applicable, explain how loss to follow-up was addressed  *Case-control study*—If applicable, explain how matching of cases and controls was addressed  *Cross-sectional study*—If applicable, describe analytical methods taking account of sampling strategy | 6-7,11 | …withdrawal from the current dialysis style or center,…were lost follow-up due to the change to other dialysis units |
|  |  |  | (*e*) Describe any sensitivity analyses | no | No sensitivity analyses |
| Results | | | | | |
| Participants | 13* | (a) Report numbers of individuals at each stage of study—eg numbers potentially eligible, examined for eligibility, confirmed eligible, included in the study, completing follow-up, and analysed | | 7,10-11 | 340 patients potentially eligible, 29 withdraw due to the incomplete data or imparity, 311 included in the study, completing follow-up number embedded in figures 3. |
|  |  | (b) Give reasons for non-participation at each stage | | 7,10-11 | …patients had kidney transplantation,… patients transferred from one type of dialysis modality to the other, and… |
|  |  | (c) Consider use of a flow diagram | | no | no |
| Descriptive data | 14* | (a) Give characteristics of study participants (eg demographic, clinical, social) and information on exposures and potential confounders | | 7-11 | Demographic characteristics, The clinical and sub-clinical comorbidity state of the dialysis patients, Dialysis related parameters, |
|  |  | (b) Indicate number of participants with missing data for each variable of interest | | 33-36 | The case number was listed in the column of tables. |
|  |  | (c) *Cohort study*—Summarise follow-up time (eg, average and total amount) | | 10 | Median follow-up (25%, 75% quartile) |
| Outcome data | 15* | *Cohort study*—Report numbers of outcome events or summary measures over time | | 10-11 | …participants died… |
|  |  | *Case-control study—*Report numbers in each exposure category, or summary measures of exposure | | */* | */* |
|  |  | *Cross-sectional study—*Report numbers of outcome events or summary measures | | */* | */* |
| Main results | 16 | (*a*) Give unadjusted estimates and, if applicable, confounder-adjusted estimates and their precision (eg, 95% confidence interval). Make clear which confounders were adjusted for and why they were included | | 11-14 | There was significant difference in…, The covariates included…, With the reference of category zero (130-160mg/dl), non-HDL categories 1-4 had higher risk of all-cause mortality (HR(95% CI):… |
|  |  | (*b*) Report category boundaries when continuous variables were categorized | | 11-14 | We classified lipids into categories as the following: TC, 0: 160-200, 1: <160, 2: …, serum potassium code (0: 3.50-5.499; 1: <3.50; 2: 5.50-5.9… |
|  |  | (*c*) If relevant, consider translating estimates of relative risk into absolute risk for a meaningful time period | | 21 | The increase in all-cause mortality rates per 1000 person-years attributable to every 1g/l decrease in ALB was… |

Continued on next page

| Other analyses | | 17 | | | Report other analyses done—eg analyses of subgroups and interactions, and sensitivity analyses | | 11-14 | Table 1 listed the biochemical comparison between the alive and died patients. The dead patients seemed to be older… |
| --- | --- | --- | --- | --- | --- | --- | --- | --- |
| Discussion | | | | | | | | |
| Key results | 18 | | Summarise key results with reference to study objectives | | | 14-15,22 | | In this cohort of subjects on stable maintenance dialysis, 54.98% patients had LDL-C level no less than 100mg/dl, while 82.91% patients with high triglycerides had non-HDL level no less than 130mg/dl. Our follow-up data supported the importance of lipids to survival…, The LDL-C 100-130mg/dl and the non-HDL 130-160mg/dl seemed to be the appropriate lipid level for dialysis patients... The demographic and biochemical characteristics comparison demonstrated that the patients in non-HDL category 130-160mg/dl had the lowest corrected calcium and calcium phosphate product… |
| Limitations | 19 | | Discuss limitations of the study, taking into account sources of potential bias or imprecision. Discuss both direction and magnitude of any potential bias | | | 21-22 | | The limitations include… |
| Interpretation | 20 | | Give a cautious overall interpretation of results considering objectives, limitations, multiplicity of analyses, results from similar studies, and other relevant evidence | | | 14-15,21-22 | | The non-HDL 130-160mg/dl seemed to be the most appropriate lipid level for dialysis patients, worthy of recommendation, because patients in this category had the lowest all-cause mortality rate... |
| Generalisability | 21 | | Discuss the generalisability (external validity) of the study results | | | 15-16,21-22 | | Our results supported those with high lipids had poor prognosis, just as in general. Meanwhile our result suggested that patients with too low lipids (LDL-C <70mg/dl, non-HDL <130mg/dl), which were recommended as ideal or optimal lipids level by guidelines, also had poor prognosis…Given the high all-cause and cardiovascular mortality rates seen in dialysis patients, the role of lipids as modifiable risk factors should be of clinical importance. The mechanism under the association of lipid and calcium need further investigation. |
| Other information | | |  | | | | | |
| Funding | | 22 | | Give the source of funding and the role of the funders for the present study and, if applicable, for the original study on which the present article is based | | | 1，23 | Funded by…, the funders’ s role is to examine and verify the application form, and mid-term review |

*Give information separately for cases and controls in case-control studies and, if applicable, for exposed and unexposed groups in cohort and cross-sectional studies.

**Note:** An Explanation and Elaboration article discusses each checklist item and gives methodological background and published examples of transparent reporting. The STROBE checklist is best used in conjunction with this article (freely available on the Web sites of PLoS Medicine at http://www.plosmedicine.org/, Annals of Internal Medicine at http://www.annals.org/, and Epidemiology at http://www.epidem.com/). Information on the STROBE Initiative is available at www.strobe-statement.org.
